# Bacterial Analogs of Plant Tetrahydropyridine Alkaloids Mediate Microbial Interactions in a Rhizosphere Model System

**DOI:** 10.1128/AEM.03058-18

**Published:** 2019-05-02

**Authors:** Gabriel L. Lozano, Hyun Bong Park, Juan I. Bravo, Eric A. Armstrong, John M. Denu, Eric V. Stabb, Nichole A. Broderick, Jason M. Crawford, Jo Handelsman

**Affiliations:** a Wisconsin Institute for Discovery and Department of Plant Pathology, University of Wisconsin–Madison, Madison, Wisconsin, USA; b Department of Molecular, Cellular and Developmental Biology, Yale University, New Haven, Connecticut, USA; c Department of Chemistry, Yale University, New Haven, Connecticut, USA; d Chemical Biology Institute, Yale University, West Haven, Connecticut, USA; e Wisconsin Institute for Discovery and Department of Biomolecular Chemistry, University of Wisconsin–Madison, Madison, Wisconsin, USA; f Department of Microbiology, University of Georgia, Athens, Georgia, USA; g Department of Molecular and Cell Biology, University of Connecticut, Storrs, Connecticut, USA; h Department of Microbial Pathogenesis, Yale School of Medicine, New Haven, Connecticut, USA; McMaster University

**Keywords:** *Flavobacterium johnsoniae*, *Pseudomonas koreensis*, antibiotics, bacterial competition, convergent evolution

## Abstract

Plants expend significant resources to select and maintain rhizosphere communities that benefit their growth and protect them from pathogens. A better understanding of assembly and function of rhizosphere microbial communities will provide new avenues for improving crop production. Secretion of antibiotics is one means by which bacteria interact with neighboring microbes and sometimes change community composition. In our analysis of a taxonomically diverse consortium from the soybean rhizosphere, we found that Pseudomonas koreensis selectively inhibits growth of Flavobacterium johnsoniae and other members of the *Bacteroidetes* grown in soybean root exudate. A genetic screen in *P. koreensis* identified a previously uncharacterized biosynthetic gene cluster responsible for the inhibitory activity. Metabolites were isolated based on biological activity and were characterized using tandem mass spectrometry, multidimensional nuclear magnetic resonance, and Mosher ester analysis, leading to the discovery of a new family of bacterial tetrahydropyridine alkaloids, koreenceine A to D (metabolites 1 to 4). Three of these metabolites are analogs of the plant alkaloid γ-coniceine. Comparative analysis of the koreenceine cluster with the γ-coniceine pathway revealed distinct polyketide synthase routes to the defining tetrahydropyridine scaffold, suggesting convergent evolution. Koreenceine-type pathways are widely distributed among *Pseudomonas* species, and koreenceine C was detected in another *Pseudomonas* species from a distantly related cluster. This work suggests that *Pseudomonas* and plants convergently evolved the ability to produce similar alkaloid metabolites that can mediate interbacterial competition in the rhizosphere.

**IMPORTANCE** The microbiomes of plants are critical to host physiology and development. Microbes are attracted to the rhizosphere due to massive secretion of plant photosynthates from roots. Microorganisms that successfully join the rhizosphere community from bulk soil have access to more abundant and diverse molecules, producing a highly competitive and selective environment. In the rhizosphere, as in other microbiomes, little is known about the genetic basis for individual species’ behaviors within the community. In this study, we characterized competition between Pseudomonas koreensis and Flavobacterium johnsoniae, two common rhizosphere inhabitants. We identified a widespread gene cluster in several *Pseudomonas* spp. that is necessary for the production of a novel family of tetrahydropyridine alkaloids that are structural analogs of plant alkaloids. We expand the known repertoire of antibiotics produced by *Pseudomonas* in the rhizosphere and demonstrate the role of the metabolites in interactions with other rhizosphere bacteria.

## Abstract (precis)

The microbiomes of plants are critical to host physiology and development. Microbes are attracted to the rhizosphere due to massive secretion of plant photosynthates from roots. Microorganisms that successfully join the rhizosphere community from bulk soil have access to more abundant and diverse molecules, producing a highly competitive and selective environment. In the rhizosphere, as in other microbiomes, little is known about the genetic basis for individual species’ behaviors within the community. In this study, we characterized competition between Pseudomonas koreensis and Flavobacterium johnsoniae, two common rhizosphere inhabitants. We identified a widespread gene cluster in several *Pseudomonas* spp. that is necessary for the production of a novel family of tetrahydropyridine alkaloids that are structural analogs of plant alkaloids. We expand the known repertoire of antibiotics produced by *Pseudomonas* in the rhizosphere and demonstrate the role of the metabolites in interactions with other rhizosphere bacteria.

## INTRODUCTION

Plants were long thought to be defined by their genes and environments. It has recently become apparent that plants are also shaped by their microbiomes, i.e., the communities of microorganisms that live on, around, and inside them (1). Microbiomes modify many environments, including humans, animals, oceans, soils, and hot springs. Comprehensive investigations of the interactions between microbiomes and their environments, as well as the interactions within microbiomes that contribute to their function and stability, are important to understanding diverse niches on Earth, including those associated with plants.

The rhizosphere comprises plant root surfaces and their surrounding soil microenvironments. Bacteria are attracted to these environments by the massive amount of plant photosynthate, in the form of sugars, organic acids, and amino acids, which is secreted from roots (2). Bacteria that colonize the rhizosphere play an essential role in plant growth and resistance to pathogens. For example, some members secrete plant hormone-like molecules, such as indole acetic acid, gibberellic acid, cytokinin, and abscisic acid, that promote plant growth (3), whereas others suppress plant diseases by secreting diverse compounds such as zwittermicin A, 2,4-diacetylphloroglucinol, and pyoluteorin (4). Thus, bacterial rhizosphere communities represent a rich reservoir of bioactive metabolites.

Use of bacteria for biological control of plant disease has been pursued for decades, but foreign microorganisms typically do not persist in native rhizosphere communities (5). Nutrient abundance, host availability, and microbial interactions define indigenous microbial community structures and limit colonization by invading bacteria. To engineer plant microbiomes to improve agricultural systems, a better understanding of the interbacterial interactions that dominate the rhizosphere is needed.

We developed the hitchhikers of the rhizosphere (THOR), a model system to examine the molecular interactions among core bacterial members of the rhizosphere (6). This model system is composed of Bacillus cereus, Flavobacterium johnsoniae, and Pseudomonas koreensis, which belong to three dominant phyla within the rhizosphere, *Firmicutes*, *Bacteroidetes*, and *Proteobacteria*, respectively. The three members display both competitive and cooperative interactions. For example, *P. koreensis* inhibits growth of *F. johnsoniae* but not in the presence of Bacillus cereus. Inhibition was only observed when bacteria were grown in soybean root exudate and is specific for members of the *Bacteroidetes*, based on testing a collection of taxonomically diverse rhizosphere bacteria (6). In this study, we characterized the genetic and molecular mechanisms by which *P. koreensis* inhibits *F. johnsoniae*. We determined that a new family of bacterial tetrahydropyridine alkaloids, designated koreenceine A to D (compounds 1 to 4), are produced by an orphan polyketide synthase (PKS) pathway and mediate inhibition of members of the *Bacteroidetes*. Koreenceine A, B, and C are structural analogs of the tetrahydropyridine alkaloid γ-coniceine and its reduced piperidine alkaloid coniine, produced by plants, and comparisons of the plant and bacterial biosynthetic pathways support a convergent evolutionary model.

## RESULTS

### Identification of an orphan *P. koreensis* pathway that is responsible for inhibiting growth of *F. johnsoniae*.

**FIG 1:**
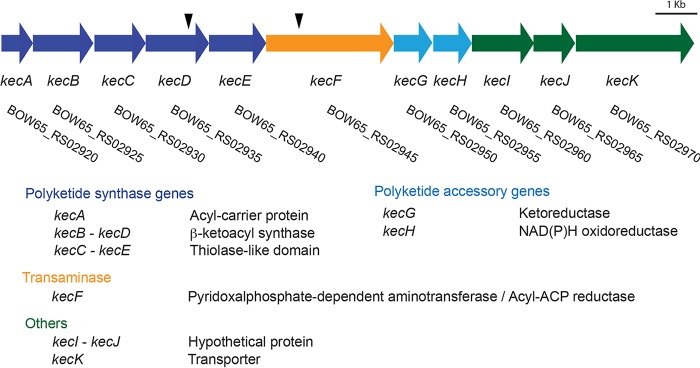
Koreenceine biosynthetic locus and the predicted function of each gene. Black arrows indicate locations of the transposons of the mutants identified.

To identify the genes required for inhibition of *F. johnsoniae* by *P. koreensis* in root exudate, we screened 2,500 *P. koreensis* transposon mutants and identified sixteen that did not inhibit *F. johnsoniae* (Table 1). Two of these mutants mapped in BOW65_RS02935 and BOW65_RS02945, which are part of an uncharacterized polyketide biosynthetic gene cluster containing 11 genes (Fig. 1). We deleted the entire gene cluster (*kecA-kecK*::Tn), which abolished inhibitory activity against *F. johnsoniae* and other members of the *Bacteroidetes* (see Fig. S1 in the supplemental material). We designated this pathway an orphan pathway, since the encoded natural product was unknown.

**TABLE 1:** *P. koreensis* mutants identified in the genetic screen with loss of inhibitory activity against *F. johnsoniae*

Mutant	ID	Predicted function	Class
1	BOW65_RS02945	Pyridoxalphosphate-dependent aminotransferase	Secondary metabolite production
2	BOW65_RS02935	3-Oxoacyl-ACP synthase	Secondary metabolite production
3, 4	BOW65_RS24575	Two-component sensor histidine kinase CbrA	Cell signaling and transcription regulation
5, 6	BOW65_RS20110	CysB family transcriptional regulator	Cell signaling and transcription regulation
7	BOW65_RS21410	Multisensor hybrid histidine kinase	Cell signaling and transcription regulation
8	BOW65_RS06475	Outer membrane protein assembly factor BamC	Cell surface
9	BOW65_RS22455	Peptidoglycan-associated lipoprotein	Cell surface
10	BOW65_RS29255	Phospholipid/glycerol acyltransferase	Cell surface
11	BOW65_RS28295	Acetylglutamate kinase	Metabolism
12	BOW65_RS08620	Methylcitrate synthase	Metabolism
13	BOW65_RS24475	Ketol-acid reductoisomerase	Metabolism
14	BOW65_RS07790	Succinyl-CoA synthetase subunit alpha	Metabolism
15	BOW65_RS08625	2-Methylisocitrate dehydratase	Metabolism
16^*a*^	BOW65_RS24600	3-Methyl-2-oxobutanoate hydroxymethyltransferase	Metabolism
16^*a*^	BOW65_RS24605	Pantoate-beta-alanine ligase	Metabolism

a Transposon insertion in the promoter 5′ region of an operon conformed for these two genes.

We developed a defined medium in which *P. koreensis* produces the gene cluster-dependent inhibitory activity against *F. johnsoniae* that was observed in root exudate (Fig. S2A). Since we identified two independent mutants in the gene encoding a sensor histidine kinase, *cbrA*, that was required for activity in root exudate (Table 1), we developed a defined medium with the goal of activating the CbrAB system (7), which controls the utilization of alternative carbon sources such as amino acids (8). Adding to defined medium the same mix of amino acids that was used to supplement root exudate induced *P. koreensis* to produce inhibitory activity in the defined medium (Fig. S2A). We next tested 19 amino acids and identified five, including aspartate, that induced inhibition of *F. johnsoniae* by *P. koreensis* (Fig. S2B). A nonhydrolyzable analog of aspartate, *N*-methyl-dl-aspartate (Asp*), did not stimulate inhibitory activity, suggesting that catabolism of certain amino acids is required for activity (Fig. S2B).

### Characterization of koreenceine metabolites from the orphan *P. koreensis* pathway.

**FIG 2:**
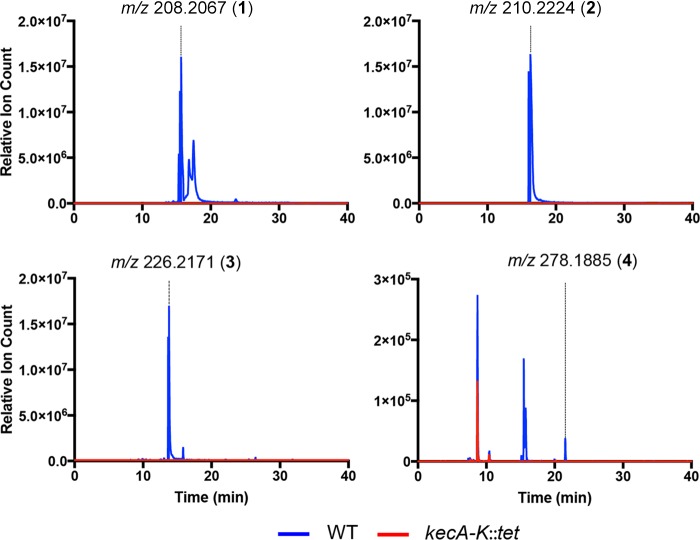
Extracted ion chromatograms from LC-HR-ESI-QTOF-MS of koreenceine A to D for the wild type and *kecA*-*kecK* deletion mutant.

To characterize the inhibitory metabolites from the orphan *P. koreensis* pathway, we compared the metabolomes of the wild-type strain and the noninhibitory mutant grown in root exudate. High-performance liquid chromatography-mass spectrometry (HPLC-MS)-based analysis of the crude organic extracts led to the identification of peaks 1 to 4 that were completely abolished in the mutant (Fig. 2). We carried out bioassay-guided preparative-scale HPLC fractionation of the crude organic extract from a culture (5 liters) of the wild-type *P. koreensis* grown in defined medium. Peaks 1, 2, and 4 were detected in fractions with antimicrobial activity against *F. johnsoniae*. High-resolution electrospray ionization-quadrupole time of flight MS (HR-ESI-QTOF-MS) data for peaks 1 to 4 revealed *m/z* 208.2067, 210.2224, 226.2171, and 278.1885, allowing us to calculate their molecular formulas as C_14_H_26_N, C_14_H_28_N, C_14_H_27_NO, and C_14_H_29_ClNO_2_, respectively (Fig. 2 and Fig. S3). We then proceeded with mass-directed isolation of these compounds from a larger-scale culture in defined medium (12 liters) of wild-type *P. koreensis* for nuclear magnetic resonance (NMR)-based structural characterization.

**FIG 3:**
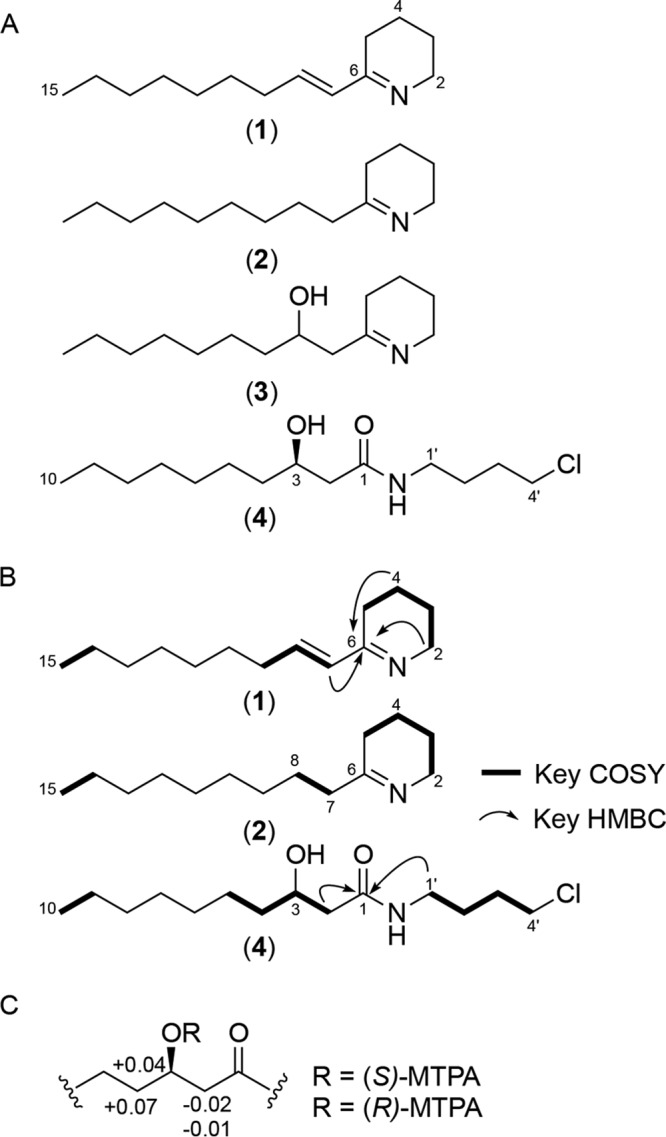
Structural characterization of koreenceines. (A) Chemical structures of compounds 1 to 4. (B) Key COSY and HMBC NMR correlations of compounds. (C) Δ*δ*_S-R_ (in ppm) for the MTPA esters of compound 4.

The chemical structures of compounds 1 to 4 were characterized through ^1^H, two-dimensional (2D)-NMR (gradient correlation spectroscopy [gCOSY], gradient heteronuclear single quantum coherence [gHSQC], and gradient heteronuclear multiple-bond coherence [gHMBC]), tandem MS, and Mosher ester analysis (Fig. 3 and Fig. S3 to S7). Briefly, ^1^H NMR spectra combined with gHSQC of peak 2 revealed the presence of six well-defined methylene groups, including one downfield-shifted signal, six additional overlapped methylene groups, and one methyl group. Consecutive COSY cross-peaks from a triplet methyl H-15 (chemical shift [*δ*_H_], 1.86) to a methylene H-7 (*δ*_H_, 2.53) established a partial structure of a nonane-like hydrocarbon chain. Additional COSY correlations from a downfield-shifted methylene H-2 (*δ*_H_, 3.53) to a methylene H-5 (*δ*_H_, 2.72) also constructed a shorter 4× CH_2_ chain. Key HMBC correlations from H-2, H-4, and H-7 to C-6 allowed us to construct the tetrahydropyridine core in compound 2. In contrast, the ^1^H NMR spectrum of compound 4 showed the presence of a hydroxyl methine H-3 (*δ*_H_, 3.94), which was evident by COSY correlations with both methylene H-2 and H-4. The connectivity between H-1ʹ (*δ*_H_, 3.20) and H-4ʹ (*δ*_H_, 3.59) was established by additional COSY correlations, which was further supported to be a 4-chlorobutanamine-like partial structure by the presence of a monochlorine isotope distribution pattern in the HR-ESI-QTOF-MS data. HMBC correlations from H-2 and H-1ʹ to an amide carbon, C-1, unambiguously constructed the chemical structure of compound 4 to be *N*-(4-chlorobutyl)-3-hydroxydecanamide. Modified Mosher’s reaction on the secondary alcohol at C-3 determined the absolute configuration of C-3 to be *R*, completing the absolute structure of compound 4. The structure of compound 1, an analog of compound 2, was elucidated based on the ^1^H and COSY NMR data that indicate the position of a *trans*-double bond between H-7 and H-8. Finally, the chemical structure of compound 3 was deduced by comparative high-resolution tandem MS analyses with the closely related compounds 1 and 2.

### Koreenceine structure-activity analysis.

We estimated the MICs of both koreenceine B (compound 2) and D (compound 4) to be 200 μg ml^−1^ against *F. johnsoniae*. We predicted that koreenceine D does not have a major role in the inhibitory activity, since koreenceine D (compound 4) is present in root exudate cultures at levels 100 times less than that of koreenceines A (compound 1), B (compound 2), and C (compound 3) (Fig. 2). We could not estimate a MIC for koreenceine A, as its levels diminish during the purification process. We synthesized koreenceine A and observed similar decomposition during purification (data not shown). Thus, we tested a semipurified fraction of koreenceine B with a trace of koreenceine A, which had a stronger inhibitory effect than koreenceine B alone (MIC of 40 μg ml^−1^). The significant increase in activity associated with trace amounts of koreenceine A suggests that this molecule is the major inhibitory molecule against *F. johnsoniae* in the THOR rhizosphere model or is synergistic with koreenceine B.

### Proposed biosynthesis of koreenceine metabolites.

**FIG 4:**
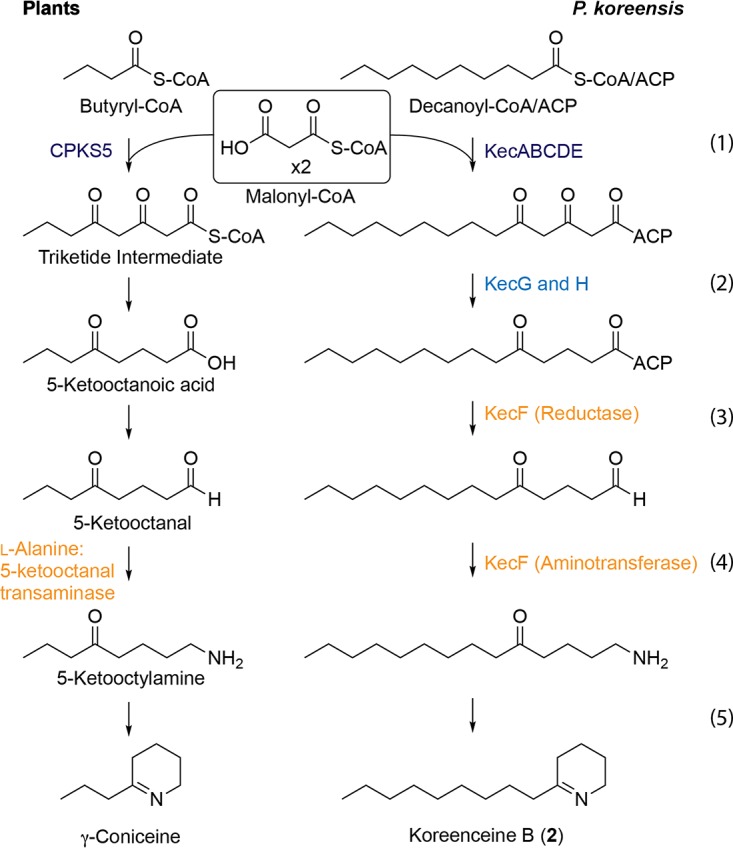
Predicted biosynthetic pathway for γ-coniceine in plants and proposed biosynthetic pathway for koreenceine B in *P. koreensis*. Similar functions are color coded to highlight the similarity between the plant pathway and the koreenceine biosynthetic locus.

Τhe defining tetrahydropyridine core of koreenceine metabolites A to C is observed in plant alkaloids such as γ-coniceine, a well-characterized alkaloid from poison hemlock (*Conium maculatum*) (9). Examination of the genes in the koreenceine biosynthetic gene cluster identified encoded proteins with homology to predicted or previously identified enzymatic activities needed for the production of γ-coniceine in plants (10–13) (Fig. 1 and 4). We propose the following biosynthetic pathway of koreenceine A to C. The first five genes of the cluster, *kecABCDE*, encode a type II polyketide synthase system: *kecA* encodes an acyl carrier protein (ACP), *kecB* and *kecD* encode β-ketoacyl synthases (KSα), and *kecC* and *kecE* encode partial β-ketoacyl synthases with conserved thiolase domains (chain length factor [CLF] or KSβ). This cluster may encode production machinery for two-heterodimer systems, KecB-KecC and KecD-KecE, for polyketide elongation with KecA and likely participate in the formation of a triketide intermediate derived from the condensation of two malonyl units and a decanoyl, 3-hydroxy-decanoyl, or *trans*-2-decenoyl unit (Fig. 4, step 1). β-Keto reductive modifications could be catalyzed by KecG and KecH reductases (Fig. 4, step 2). Aminotransferase KecF is predicted to catalyze transamination of the aldehyde intermediate facilitating tetrahydropyridine cyclization. KecF appears to be a multidomain protein with a predicted aminotransferase at the N terminus and a general NAD(P)-binding domain (EMBL-EBI accession no. IPR036291) and a conserved protein domain, COG5322, at the C terminus. Interestingly, long-chain fatty acyl-ACP reductases from *Cyanobacteria* share these features and generate fatty aldehydes from the reduction of fatty acid intermediates bound to ACP (14). We predict that KecF reduces the ACP-polyketide intermediate to a polyketide aldehyde with the C-terminal domain (Fig. 4, step 3), followed by transamination by the N-terminal domain (Fig. 4, step 4). Finally, the amine intermediate could undergo a nonenzymatic cyclization, as observed in γ-coniceine (Fig. 4, step 5) (12). We predict that koreenceine D is derived from koreenceine C by an unidentified halogenase reaction, as analogous metabolite sets have been detected in plants (Fig. S8) (15). The last three genes, *kecIJK*, may participate in the translocation of the koreenceine alkaloids outside the cell. KecI and KecJ are hypothetical proteins predicted to localize in the membrane, and KecK has homology with membrane-bound drug transporters.

### Convergent evolution of pathways for production of γ-coniceine-like alkaloids in plants and *P. koreensis*.

The biosynthetic pathway for production of γ-coniceine is still under investigation, but ^14^C feeding experiments in *C. maculatum* coupled with chemical degradation of the labeled products suggest that γ-coniceine is not derived from an amino acid like other plant alkaloids; rather, it is derived from a polyketide chain produced by the condensation of acetate units (10). Type III polyketide synthases common in plants are iterative homodimers that orchestrate the acyl-CoA-mediated priming, extension, and cyclization reactions for polyketide products without the use of acyl carrier proteins (16). Recently, Hotti et al. found CPKS5, a non-chalcone synthase/stilbene synthase (CHS/STS)-type III polyketide synthase expressed in tissues that contain γ-coniceine (Fig. 4) (11). The pathway that we identified in *P. koreensis* encodes two type II PKS systems involved in production of the proposed polyketide intermediate (Fig. 1). Although the pathway that we identified in *P. koreensis* produces compounds related to the plant alkaloids, the PKSs from the plant and bacterial kingdoms share little similarity. We propose that convergent evolution led to two different polyketide pathways for the production of γ-coniceine-like metabolites in plants and bacteria.

### Distribution of the koreenceine cluster.

**FIG 5:**
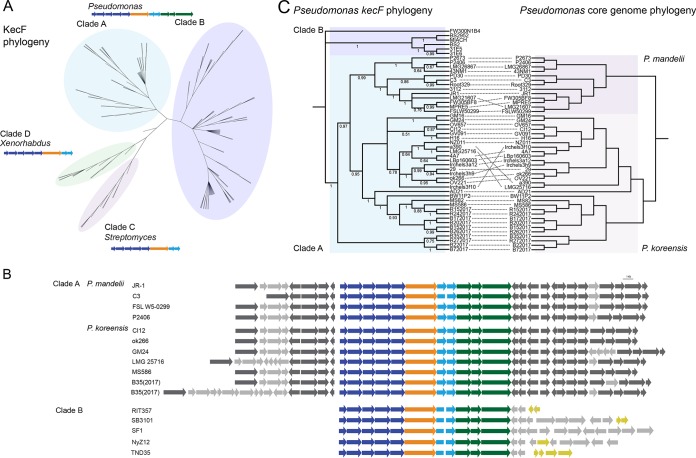
Phylogenetic analysis of the koreenceine biosynthetic locus and its distribution across bacteria. (A) ML phylogenetic tree estimated from the amino acid sequence of KecF and the corresponding structure of the koreenceine-like gene cluster present in each clade. (B) Schematic representation of the koreenceine-like biosynthetic locus and its genomic context from several *Pseudomonas* spp. from clade A and clade B. Genes conserved in all genomes from *P. mandelii* and *P. koreensis* are in dark gray, and variable or unique genes are in gray. Genes that likely experienced horizontal gene transfer events are in yellow. (C) Comparison of the phylogenies of *kecF* genes and their associated *Pseudomonas* genomes belonging to clade A *kecF* homologues. Both phylogenies correspond to ML analyses of the nucleotide sequence of the genes or the core genome. The dotted lines connect the cluster with its corresponding *Pseudomonas* genome.

Similar koreenceine-like clusters have previously been identified by functional screens for antimicrobial activities (17, 18); however, there are no reports of the metabolites being produced. We identified 179 koreenceine-like clusters in genomes in the NCBI database (June 2018). The majority of these clusters are in *Pseudomonas* genomes, although we found some partial clusters in *Xenorhabdus* and *Streptomyces* species genomes (Fig. 5A). We used maximum-likelihood analysis of the amino acid sequence of the aminotransferase-reductase protein, KecF, as a representative of the koreenceine cluster for phylogenetic reconstruction. We observed four main clades that are each associated with a bacterial genus (Fig. 5A). Clades A and B, which contain 93% of the clusters, are found in *Pseudomonas* genomes. The koreenceine gene cluster identified in *P. koreensis* in this study belongs to clade A; other clade A clusters are located in the same genomic context in P. koreensis and P. mandelii, two closely related species in the P. fluorescens complex (19) (Fig. 5B). Clades C and D were found in *Streptomyces* and *Xenorhabdus* spp., respectively, although the *kecI*, *kecJ*, and *kecK* genes are missing.

Clade A and the *P. koreensis* and *P. mandelii* genomes are phylogenetically parallel (Fig. 5C). We hypothesize that the gene cluster was acquired before Pseudomonas fluorescens complex diversification of *P. koreensis* and *P. mandelii* from a common ancestor. We also hypothesize that the pathway was maintained in these *Pseudomonas* spp. by vertical transmission. In contrast, clade B contains clusters present in P. putida and several species of the P. fluorescens complex in which there is no conservation in genome localization, and the clusters are frequently associated with diverse elements that mediate horizontal gene transfer, such as transposases, integrases, and tRNAs (Fig. 5B). This suggests different strategies to maintain koreenceine-type gene clusters in diverse *Pseudomonas* species.

Another *Pseudomonas* isolate (SWI36) was reported to inhibit B. cereus. Its activity was dependent on a koreenceine-type gene cluster from clade B (18), but we found that the strain did not inhibit *F. johnsoniae*. Under certain conditions, *P. koreensis* inhibited B. cereus, and the activity was dependent on the koreenceine cluster, suggesting a similarity with the SWI36 cluster (Fig. S9). Indeed, targeted metabolomic analysis of *Pseudomonas* sp. SWI36 cell-free culture detected koreenceine C (Fig. S10). These data suggest that different koreenceine-like gene clusters in *Pseudomonas* genomes have the capacity to synthesize koreenceine metabolites.

## DISCUSSION

In this work, we aimed to understand the molecular basis for the growth inhibition of *F. johnsoniae* by *P. koreensis* on a route to elucidating interactions within the rhizosphere microbiome. We have shown that *P. koreensis* inhibits *F. johnsoniae* growth through the production and secretion of novel secondary metabolites, koreenceine A to D (metabolites 1 to 4), which have structural similarity to the plant metabolite γ-coniceine. Based on the biosynthetic gene cluster identified through our genetic screen, we propose a type II polyketide biosynthetic pathway for these bacterial alkaloids. Classical type II polyketide synthases are iterative heterodimer systems. It is currently unclear if the two heterodimer systems present in the biosynthetic cluster act in a modular or iterative manner, but the number of putative β-ketoacyl synthase genes in the pathway is consistent with modular biosynthesis. Plants also use a polyketide pathway mediated by an iterative type III polyketide synthase, providing a new example of convergent evolution between these organisms for the synthesis of related alkaloids. The tetrahydropyridine core of the koreenceine metabolites is found in well-known plant alkaloids, such as the active cytotoxin γ-coniceine from poison hemlock (*C. maculatum*). Thus, koreenceine alkaloids may play roles in interbacterial and interdomain communication or inhibition that changes the rhizosphere community structure.

Members of the genus *Pseudomonas* are ubiquitous in nature and thrive in soil, on plants, and on moist surfaces. *P. koreensis* and other members of the Pseudomonas fluorescens complex are often studied for their capacity to colonize the rhizosphere and protect plants from pathogens. Previous research demonstrated that P. fluorescens suppresses plant disease through production of phenazine-1-carboxylic acid (PCA), which targets the fungal pathogen Gaeumannomyces graminis (20). P. fluorescens also produces a suite of antimicrobial compounds, including 2,4-diacetylphloroglucinol, pyoluteorin, pyrrolnitrin, lipopeptides, and hydrogen cyanide (4), and members of the P. fluorescens complex also produce plant hormones, such as indole acetic acid and gibberellic acid, that stimulate plant growth. In this paper, we expand the known repertoire of metabolites from the P. fluorescens complex with discovery of koreenceine A to D. Unlike most of the P. fluorescens metabolites that inhibit fungal pathogens, the koreenceines mediate interactions between *P. koreensis* and diverse members of the *Bacteroidetes*, including *F. johnsoniae*, in a family-specific manner (6). Competition between members of the P. fluorescens complex and *Flavobacterium* spp. in natural settings has been reported; *in vivo* studies showed a selective reduction of *Flavobacterium* spp. in the Arabidopsis thaliana rhizosphere when *Pseudomonas* sp. CH267 was added to soil (21). Together, these results highlight the relevance of characterizing bacterium-bacterium interactions in the rhizosphere.

We identified a gene cluster necessary for the production of the koreenceine metabolites. Other koreenceine-like clusters have been predicted to mediate (22) and others are associated with (17, 18) antagonistic activity against diverse microorganisms. Thirty-five percent of *P. koreensis* and *P. mandelii* genomes in the NCBI database contain this gene cluster, and there are at least 160 *Pseudomonas* genomes harboring a related cluster, indicating the widespread nature of this pathway among *Pseudomonas* spp. Despite its ubiquity, structural characterization of the products of the biosynthetic gene cluster remained undefined until this study.

Bioinformatic analysis of the proposed activities of the genes in the cluster enabled us to propose a biosynthetic pathway for the formation of a C_14_ polyketide with a tetrahydropyridine-type ring from a noncanonical type II PKS system (Fig. 2). In bacteria, tetrahydropyridine-type rings could be derived from lysine cyclization (23), as observed in plants, or by a two-step reduction-transamination route of polyketide intermediates (24). We propose that the multidomain protein KecF directs both steps, reduction of the acyl intermediate to generate the acyl-aldehyde and transamination. This differs from the established route, in which the two activities are encoded by different genes, and the reductase domain represents the terminal domain of a type I polyketide synthase (Fig. 4) (24). We propose that β-ketoacyl synthase(s) incorporates *trans*-2-decenoyl, decanoyl, or 3-hydroxy-decanoyl units to generate koreenceine A, B, or C, respectively. It is unclear if these acyl units are recruited as coenzyme A (CoA) esters from the β-oxidation pathway or as ACP esters from fatty acid synthesis, although the *R* configuration in koreenceine D suggests substrate sampling from the fatty acid synthesis pool (i.e., fatty acyl-ACP).

Koreenceine A to C share structural features with γ-coniceine, the metabolite responsible for the toxicity of the poison hemlock, a plant once used in death sentences and the means by which Socrates took his own life after receiving such a sentence (399 BCE). The structural similarity is the result of convergent evolution and suggests additional functionality of the metabolites. The koreenceines might play a protective role similar to that of the hemlock toxins, protecting plant roots from invertebrate pests by poisoning their nervous systems (9), or the koreenceines might alter plant development, since γ-coniceine is thought to be a plant hormone (9, 25). Thus, future work will focus on characterizing the effect of koreenceine A to C on plant development and protection.

## MATERIALS AND METHODS

### Bacterial strains and culture conditions.

*F. johnsoniae* CI04, *P. koreensis* CI12, B. cereus UW85, *Pseudomonas* sp. SWI36, Flavobacterium johnsoniae CI64, *Chryseobacterium* sp. CI02, *Chryseobacterium* sp. CI26, *Sphingobacterium* sp. CI01, and *Sphingobacterium* sp. CI48 were propagated on 1/10-strength tryptic soy agar and grown in liquid culture in half-strength tryptic soy broth (TSB) at 28°C with vigorous shaking.

### Production of root exudates and defined media.

Soybean seeds were surface sterilized with 6% sodium hypochlorite for 10 min, washed with sterile deionized water, transferred to water agar plates, and allowed to germinate for 3 days in the dark at 25°C. Twelve seedlings were grown in a hydroponic system using 250 ml of modified Hoagland's plant growth solution (26), which was collected after 10 days of plant growth in an environmental chamber (12-h photoperiod, 25°C), filter sterilized, and stored at −20°C until used as root exudate. A defined medium was based on basal salt medium [1.77 g liter^−1^ Na_2_HPO_4_, 1.70 g liter^−1^ KH_2_PO_4_, 1.00 g liter^−1^ (NH_4_)_2_SO_4_, 0.16 g liter^−1^ MgCl_2_·6H_2_O, 5.00 mg liter^−1^ FeSO_4_·7H_2_O]. A carbon source (pyruvate, mannitol, or glucose) was added to a final concentration of 4 mM. An amino acid mix of equal parts alanine, aspartate, leucine, serine, threonine, and valine was added to the root exudate or the defined medium at a final concentration of 6 mM. Individual amino acids and *N*-methyl-dl-aspartate were also added to a final concentration of 6 mM.

### *P. koreensis* mutant library generation by transposon mutagenesis.

*P. koreensis* CI12 and Escherichia coli S17-1λpir with pSAM_BT20 (27) supplemented with ampicillin (100 μg ml^−1^) were first grown individually for 16 h in LB at 28°C and 37°C, respectively, with agitation. Cells were washed and resuspended in LB to an optical density at 600 nm of 2.0. One volume of E. coli S17-1λpir with pSAM_BT20 was mixed with two volumes of *P. koreensis* CI12. Cells were harvested (6,000 × *g*, 6 min), resuspended in 100 μl of LB, and spotted on LBA. Plates were incubated at 28°C for 16 h. Each conjugation mixture was scraped off the plate and resuspended in 2.5 ml of LB, and 350-μl aliquots were plated on LB containing gentamicin (50 μg ml^−1^) and chloramphenicol (10 μg ml^−1^) to select for *P. koreensis* CI12 transconjugants. Plates were incubated for 2 days at 28°C.

### Genetic screen of *P. koreensis* mutants defective in inhibitory activity.

*P. koreensis* CI12 mutants were grown for 16 h in 96-deepwell plates filled with half-strength TSB, covered with sterile breathable sealing films, and incubated at 28°C with agitation. For each plate, the first well was inoculated with wild-type *P. koreensis* CI12 and the last well was left without *P. koreensis* CI12. *F. johnsoniae* CI04 was grown and washed as described above. Root exudate was inoculated with ∼10^7^ *F. johnsoniae* CI04 cells per ml, and 200-μl aliquots were added to each well of 96-well microplates. Two μl from each mutant *P. koreensis* CI12 culture was transferred to the corresponding wells on the microplates, which were then covered by sealing films and incubated at 28°C with slight agitation for 2 days. Five μl from each well was then spotted on Casitone-yeast extract agar (CYE) (10 g liter^−1^ Casitone, 5 g liter^−1^ yeast extract, 8 mM MgSO_4_,10 mM Tris buffer, 15 g liter^−1^ agar) containing kanamycin (10 μg ml^−1^) to select for *F. johnsoniae*, and plates were incubated at 28°C for 2 days. In the wild-type control, no *F. johnsoniae* colonies were detected in the spot. Mutants that did not inhibit *F. johnsoniae*, which grew in the spot from mutant cultures, were streaked on a second plate for further analysis. The loss of inhibitory activity of candidate *P. koreensis* mutants was verified in a second coculture, and mutant growth was then compared to wild-type growth to rule out candidates that failed to inhibit *F. johnsoniae* due to their own growth deficiency.

### Location transposons in *P. koreensis* mutants defective in *F. johnsoniae* inhibition.

For each mutant, 1 ml of liquid culture grown for 16 h was harvested (6,000 × *g*, 6 min), and cells were resuspended in 400 μl of TE (10 μM Tris-HCl, pH 7.4; 1 μM EDTA, pH 8.0). Samples were boiled for 6 min and centrifuged (6,000 × *g*, 6 min), and 2 μl of supernatant was used as a template for DNA amplification. Transposon locations were determined by arbitrarily primed PCR, which consisted of a nested PCR using first-round primer GenPATseq1 and either AR1A or AR1B and second-round primer GenPATseq2 and AR2 (Table 2). PCR products from the second round were purified by gel extraction (QIAquick gel extraction kit; Qiagen) and then sequenced using primer GenPATseq2. Genomic regions flanking the transposons were identified by comparing the sequences to the genome using the nucleotide BLAST alignment tool.

**TABLE 2:** Primers used in this study

Name	Sequence
GenPATseq1	CTTGGATGCCCGAGGCATAG
GenPATseq2	CTGTACAAAAAAACAGTCATAACAAGCCATG
AR1A	GGCCACGCGTCGACTAGTACNNNNNNNNNNGTAAT
AR1B	GGCCACGCGTCGACTAGTACNNNNNNNNNNGATGC
AR2	GGCCACGCGTCGACTAGTAC
mutSGCA_For	CACCCGCAAGCCTGCAATAGACGGAC
mutSGCA_Rev	CCTGTCGTCTCAGGAAAGGTGCGGTACCTTCTATCTCCCTATATGTCGTGAC
mutSGCB_For	GTCACGACATATAGGGAGATAGAAGGTACCGCACCTTTCCTGAGACGACAGG
mutSGCB_Rev	GCACCTGACATTCGTCTATCCGATC
TetA_For	CACCGGTACCTCCTCCAAGCCAGTTACCTCGG
TetA_Rev	GGTACCTGCTCAGGTCGCAGACGTTTTG
pJN105Mob_For	TAGGCGCGCCTGTGGTCAAGCTCGTGGGC
pJN105Mob_Rev	CACCGGCGCGCCCAATTCGTTCAAGCCGAGATCGGC

### Chromosomal deletion of the koreenceine gene cluster in *P. koreensis*.

The koreenceine cluster was deleted by allelic exchange and replaced with a tetracycline resistance cassette. The *kecA*-*kecK* deletion cassette was constructed by a modified version of an overlap extension (OE) PCR strategy. Fragments 1 kb upstream and 1 kb downstream of the *kecA* to *kecK* genes were amplified using primers mutSGCA_For/mutSGCA_Rev and mutSGCB_For/mutSGCB_Rev respectively (Table 2). The PCR products were cloned in pENTR/d-TOPO, generating pkecA-K_ENTR. Primers mutSGCA_Rev and mutSGCB_For were designed to include a KpnI site in their overlapping region to allow introduction of a resistance gene. A tetracycline resistance cassette was amplified from pACYC184 using primers TetA_For/TetA_Rev, which contain KpnI sites in the 5′ region, and cloned into pENTR/d-TOPO to generate pTetA_ENTR. A *mob* element was amplified from pJN105 using primers pJN105Mob_For/pJN105Mob_Rev (Table 2), in which an AscI site in the 5′ region was added, and cloned in pENTR/d-TOPO, generating pmob_ENTR. The tetracycline resistance cassette was recovered from pTetA_ENTR using KpnI and cloned between the region upstream and downstream of pkecA-K_ENTR, and the *mob* element was recovered from pmob_ENTR using AscI and cloned into an AscI site in the pENTR backbone, generating pkecA-K_TetA_mob_ENTR. Conjugation mixtures of *P. koreensis* CI12 and E. coli S17-1λpir carrying the pkecA-K_TetA_mob_ENTR vector were prepared by following the procedure for transposon mutant generation. Double recombinant *P. koreensis* CI12 transconjugants were selected by their ability to grow on tetracycline (10 μg ml^−1^) and inability to grow on kanamycin (50 μg ml^−1^). The *kekA-kekK* deletion mutant was confirmed by PCR using primers mutSGCA_For and mutSGCB_Rev. The *kekA-kekK* deletion mutant was further confirmed by evaluating growth of *F. johnsoniae*, and other members of the *Bacteroidetes*, in its presence.

### General information for the analysis and identification of metabolites.

^1^H and 2D (gCOSY, gHSQC, and gHMBC) NMR spectra were obtained on an Agilent (USA) 600-MHz NMR spectrometer with a cold probe, and the chemical shifts were recorded as *δ* values (ppm) with methanol-*d*_4_ as the standard NMR solvent. Materials were routinely analyzed on an Agilent 6120 single-quadrupole liquid chromatography-mass spectrometry (LC-MS) system (Phenomenex Kinetex C_18_ column, 250 by 4.6 mm, 5 μm; flow rate, 0.7 ml min^−1^; mobile phase composition, H_2_O and acetonitrile (ACN) containing 0.1% trifluoroacetic acid (TFA); method, 0 to 30 min with 10% to 100% ACN, hold for 5 min with 100% ACN, hold for 1 min with 100% to 10% ACN). High-resolution electrospray ionization mass spectrometry (HR-ESI-MS) data were obtained using an Agilent iFunnel 6550 QTOF mass spectrometer fitted with an ESI source coupled to an Agilent (USA) 1290 Infinity HPLC system. Open-column chromatography was carried out on a Waters Sep-Pak Vac 35-cc (10-g) C_18_ column. Metabolite isolations were performed using an Agilent (USA) Prepstar HPLC system with an Agilent (USA) Polaris C_18_-A 5-μm (21.2- by 250-mm) column, a Phenomenex (USA) Luna C_18_(2) (100-Å) 10-μm (10.0- by 250-mm) column, a Phenomenex (USA) Luna C_18_(2) (100-Å) 10-μm (10.0- by 250-mm) column, and an Agilent Polaris 5 Amide-C_18_ (250- by 10.0-mm) column.

### Isolation of metabolites.

*P. koreensis CI12* was grown in defined medium with pyruvate as the carbon source and supplemented with the amino acid mix or 3 mM glutamate for 3 days. Crude extract was generated by liquid-liquid extraction using one volume of 2-butanol per one volume of filter supernatant and dried by rotary evaporation. The crude extracts (400 mg) from the 12-liter culture supernatant were resuspended in water and methanol (1:1 ratio), adsorbed onto Celite 110, and dried by rotary evaporation. The resulting powdery materials were loaded on the Waters Sep-Pak Vac 35-cc (10-g) C_18_ cartridge, and the metabolites were separated by solvent fractionation, eluting with a step gradient from 20% to 100% aqueous methanol to yield five subfractions (20%, 40%, 60%, 80%, and 100% methanol containing 0.1% TFA). Reverse-phase LC-MS analysis (10% to 100% aqueous acetonitrile in 0.1% TFA, 30-min gradient) revealed that the 60% fraction included both molecules 1 and 2, and the fraction was dried under reduced pressure. This fraction (60 mg) was then separated by reverse-phase HPLC equipped with an Agilent Polaris C_18_-A 5-μm (21.2- by 250-mm) column with an isocratic solvent system (50% acetonitrile in water and 0.1% TFA over 20 min, 8 ml min^−1^, 1-min fraction collection interval). Compound 1 from the pooled fraction (11 and 12) (retention time [*t*_R_] = 25.3 min, 0.2 mg) was partially purified over the Phenomenex Luna C_18_(2) 10-μm (10.0- by 250-mm) column with a linear gradient elution (20% to 80% acetonitrile in water and 0.1% TFA over 30 min). The combined HPLC fraction (11 and 12) was subsequently purified by reverse-phase HPLC [Phenomenex Luna C_18_(2) 10-μm (10- by 250-mm) column] with a linear gradient elution (20% to 80% acetonitrile in water and 0.1% TFA over 30 min) to yield pure compound 2 (*t*_R_ = 25.8 min, 1.2 mg). Compound 4 was detected in the 80% aqueous methanol Sep-Pak fraction and was separated over an Agilent Polaris C_18_-A 5-μm (21.2- by 250-mm) column (flow rate, 8.0 ml/min; gradient elution, 10% to 100% aqueous acetonitrile in 0.1% TFA for 30 min, 1-min fraction collection). HPLC fraction 24 was then separated over the Phenomenex Luna C_18_(2) 10-μm (10- × 250-mm) column with 50% to 100% acetonitrile in water containing 0.1% TFA over 30 min at a flow rate of 4 ml min^−1^, followed by being subjected to an Agilent Polaris 5 Amide-C_18_ (250- by 10-mm) column with the same elution system (flow rate, 4 ml min^−1^; purification method, 50% to 100% acetonitrile in water containing 0.1% TFA over 30 min) to yield pure compound 4 (*t*_R_ = 9.43 min, 0.7 mg).

**(i) (*E*)-6-(non-1-en-1-yl)-2,3,4,5-tetrahydropyridine (compound 1).** Colorless solid; ^1^H NMR (CD_3_OD, 600 MHz) *δ* 7.21 to 7.12 (1H, m, H-8), 6.35 (1H, d, *J *=* *16.0 Hz, H-7), 3.59 (2H, m, H-2), 2.91 (2H, m, H-5), 2.30 (2H, m, H-9), 1.77 (2H, m, H-3), 1.71 (2H, m, H-4), 1.42 (2H, m, H-10), 1.27 to 1.20 (8H, m, H-11, H-12, H-13, H-14), 0.84 (3H, t, *J *=* *7.0 Hz, H-15); HR-ESI-QTOF-MS [M + H]^+^ *m/z* 208.2067 (calculated for C_14_H_26_N, 208.2065).

**(ii) 6-Nonyl-2,3,4,5-tetrahydropyridine (compound 2).** Colorless solid; ^1^H NMR (CD_3_OD, 600 MHz) *δ* 3.53 (2H, t, *J *=* *5.5 Hz, H-2), 2.72 (2H, t, *J *=* *6.1 Hz, H-5), 2.53 (2H, m, H-7), 1.73 (2H, m, H-3), 1.68 (2H, m, H-4), 1.54 (2H, dt, *J *=* *14.6, 6.8 Hz, H-8), 1.28-1.15 (12H, m, H-9, H-10, H-11, H-12, H-13, H-14), 0.83 (3H, t, *J *=* *7.0 Hz, H-15), ^13^C NMR (CD_3_OD, 125 MHz) *δ* 192.1 (C-6), 44.4 (C-2), 37.7 (C-7), 31.6 (C-13), 29.4 (C-5), 28.0 to 29.0 (C-9, C-10, C-11, C-12), 25.5 (C-8), 22.5 (C-14), 19.2 (C-3), 16.8 (C-4), 14.4 (C-15); HR-ESI-QTOF-MS [M + H]^+^ *m/z* 210.2224 (calculated for C_14_H_28_N, 210.2222).

**(iii) (*R*)-*N*-(4-Chlorobutyl)-3-hydroxydecanamide (compound 4).** Colorless solid; ^1^H NMR (CD_3_OD, 600 MHz) *δ* 3.94 (1H, m, H-3), 3.59 (2H, t, *J *=* *6.5 Hz, H-4ʹ), 3.20 (2H, m, H-1ʹ), 2.27 (2H, m, H-2), 1.78 (2H, m, H-3ʹ), 1.63 (2H, dt, *J *=* *14.3, 7.0 Hz, H-2ʹ), 1.43 (2H, m, H-4), 1.36 to 1.25 (10H, m, H-5, H-6, H-7, H-8, H-9), 0.90 (3H, t, *J *=* *6.9 Hz, H-10), ^13^C NMR (CD_3_OD, 125 MHz) *δ* 172.6 (C-1), 68.2 (C-3), 44.1 (C-4ʹ), 43.8 (C-2), 37.9 (C-1ʹ), 36.8 (C-4), 31.8 (C-8), 29.5 (C-3ʹ), 29.4 (C-5 or C-6, C-7), 26.3 (C-2ʹ), 22.6 (C-5 or C-6, C-9), 13.4 (C-10); HR-ESI-QTOF-MS [M + H]^+^ *m/z* 278.1885 (calculated for C_14_H_29_ClNO_2_, 278.1887).

### Determination of absolute configuration of metabolite 4.

The absolute configuration of metabolite 4 was determined using the modified Mosher’s method with *R*- and *S*-*α*-methoxy-(trifluoromethyl)phenylacetyl chloride (MTPA-Cl) (28). Compound 4 (0.5 mg) was prepared in two vials (0.25 mg), and each sample was dissolved in 250* *μl of dried pyridine-*d*_5_ in vials purged with N_2_ gas. Dimethylaminopyridine (DMAP) (0.5 mg) was added to both vials, followed by the addition of 5* *μl of *S*- and *R*-MTPA-Cl solution (2%, vol/vol) at room temperature. After 18 h, the reaction mixtures were dried under reduced pressure. ^1^H NMR spectra of the Mosher esters (*S*-MTPA ester and *R*-MTPA ester) were collected in methanol-*d*_4_, and the chemical shift differences of the Mosher esters of compound 4 were calculated as Δ*δ*_S-R_.

### Characterization of *Pseudomonas* sp. SWI36.

*Pseudomonas* sp. SWI36 and *P. koreensis* inhibitory interactions against B. cereus and *F. johnsoniae* were evaluated with a modified spread-patch method. Strains were grown separately for 20 h. One-ml aliquots of cultures of each strain were centrifuged (6,000 × *g*, 6 min) and resuspended in 1 ml of the same medium (undiluted cultures), and a 1:100 dilution of B. cereus and *F. johnsoniae* was prepared in the same medium (diluted culture). Nutrient agar plates were spread with 100 μl of either B. cereus or *F. johnsoniae* diluted cultures and spotted with 10 μl of the undiluted cultures of *Pseudomonas* sp. SWI36 and *P. koreensis*. Plates were then incubated at 28°C and inspected for zones of inhibition after 2 days. Crude extract of *Pseudomonas* sp. SWI36 and *Pseudomonas* sp. SWI36 *kecF*::Tn culture in nutrient broth were prepared as described above. Extracted materials were analyzed on an LC-MS system consisting of a Thermo Fisher Scientific (Waltham, MA) Q Exactive Orbitrap mass spectrometer with an ESI source coupled to a Vanquish UHPLC (Thermo Accucore Vanquish C_18_ column, 100 by 2.1 mm, 1.5 μm; flow rate, 0.2 ml min^−1^; mobile phase composition, H_2_O and ACN containing 0.1% TFA; method, 0 to 1 min with 10% ACN, 1 to 4 min with 10% to 35% ACN, 4 to 12 min with 35% to 70% ACN, 12 to 16 min with 70% to 98% ACN, 16- to 20-min hold with 98% ACN, 20 to 21 min with 98% to 10% ACN, and 21 to 23 min with 10% ACN). MS1 scans were acquired with positive ionization over an *m/z* range of 188 to 1,275 with settings of 1e−6 AGC, 100-ms maximum integration time, and 70-K resolution.

### Phylogenetic analysis.

Genetic regions with homology to the koreenceine biosynthetic cluster were identified by BLAST alignment tools (29) using the *P. koreensis* CI12 KecF protein sequence in the NCBI database. All of the KecF homologues identified were part of koreenceine-like clusters (genomes harboring these are listed in Table S1 in the supplemental material). Protein and nucleotide sequence alignments of *kecF* were performed with MAFFT, version 7 (30), and were manually adjusted using as a guide the residue-wise confidence scores generated by GUIDANCE2 (31). Best-fit models of amino acid or nucleotide replacement were selected. Evolutionary analyses were inferred by maximum likelihood (ML) methods conducted in MEGA X (32). The *P. koreensis* and *P. mandelii* phylogenomic reconstruction was done by phylogenetic and molecular evolutionary (PhaME) analysis software (33). PhaME identified single-nucleotide polymorphisms from the core genome alignments, and the phylogenetic relationships were inferred by ML using FastTree. Phylogenetic trees were visualized using the interactive tree of life (iTOL) (34).

## Supplementary Material

Supplemental file 1
