# Supplementary material for: Bacterial Analogs of Plant Tetrahydropyridine Alkaloids Mediate Microbial Interactions in a Rhizosphere Model System
Source: Appl Environ Microbiol. 2019 May 2;85(10):e03058-18. doi: 10.1128/AEM.03058-18 (PMC6498172; doi:10.1128/AEM.03058-18)
Supplement: Supplemental file 1 [file AEM.03058-18-s0001.pdf]

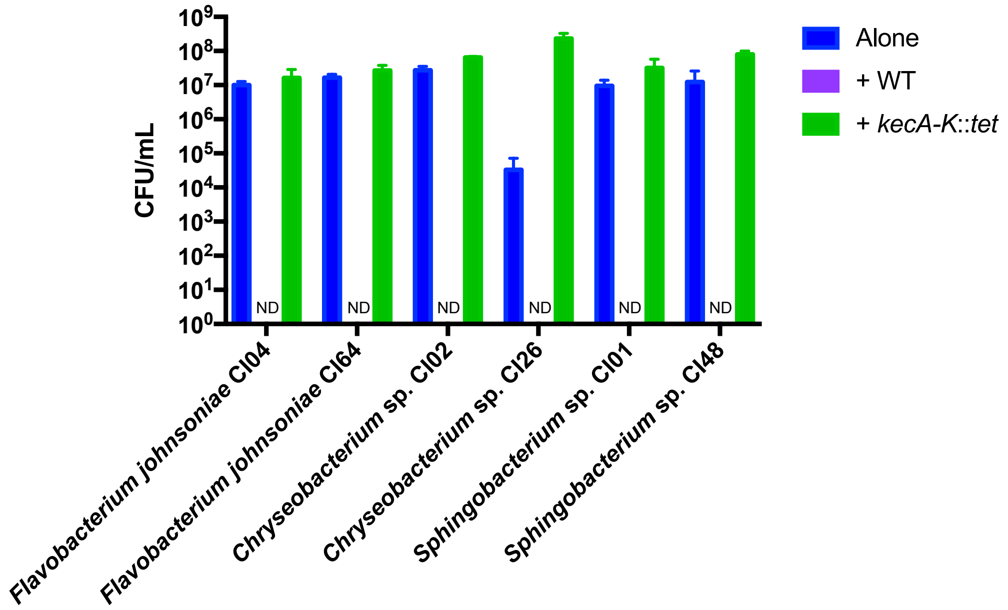

FIG S1. Evaluation of the role of the koreenceine gene cluster in inhibition of *F. johnsoniae* and other members of the Bacteroidetes. Population of several Bacteroidetes members isolated from the soybean rhizosphere when grown alone, with *P. koreensis* wild type, or *P. koreensis kecA-K*::*tet*. ND, not detected. Error bars represent ± Standard deviation.

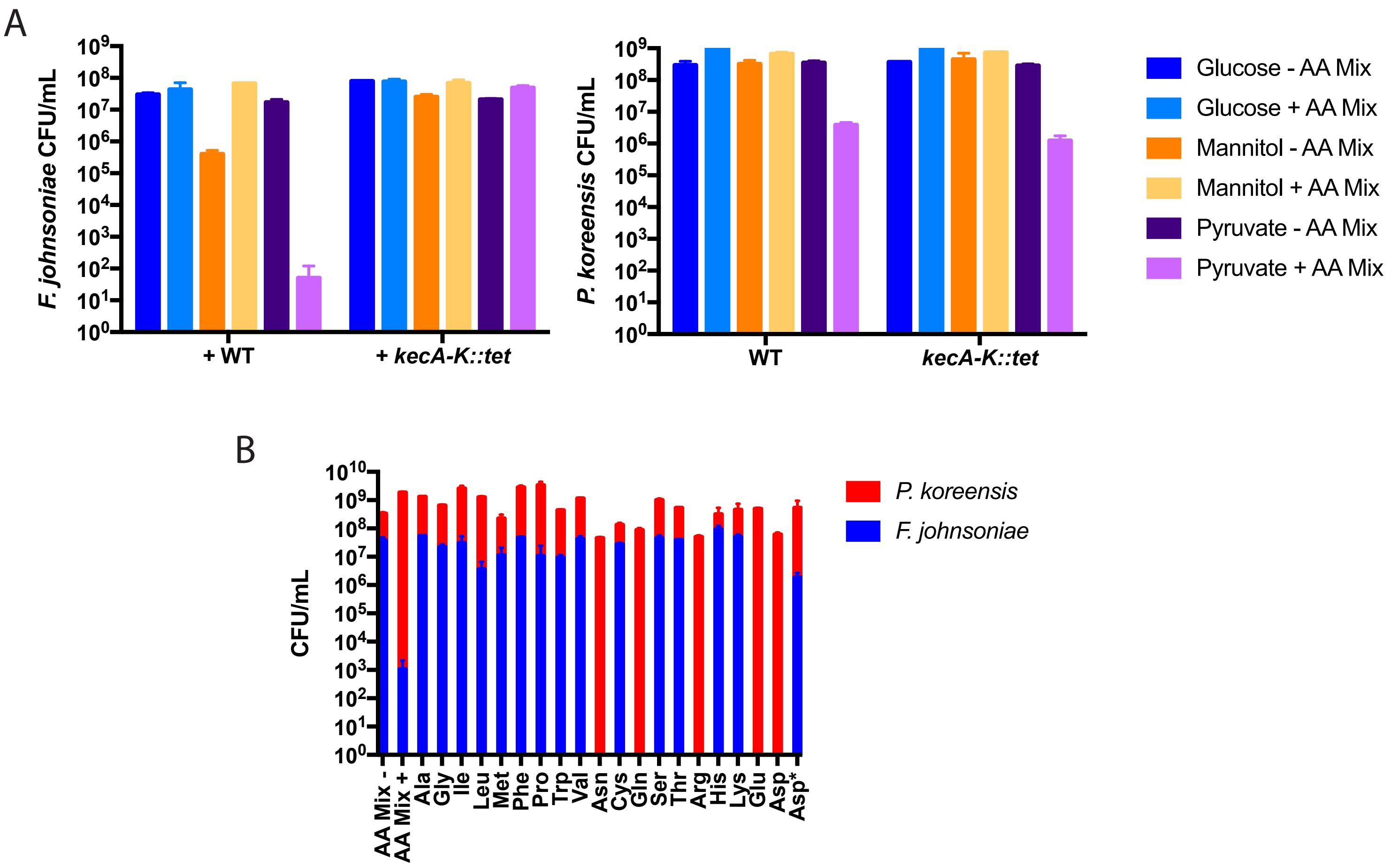

FIG S2. Development of a defined medium that supports the ability of *P. koreensis* to inhibit *F. johnsoniae*. A) Population of *P. koreensis* and *F. johnsoniae* in co-culture in a defined medium with glucose, mannitol or pyruvate as carbon sources with or without an amino acid mixture. B) Population of *P. koreensis* and *F. johnsoniae* in co-culture in a defined medium with or without an amino acid mixture, or a single amino acid, or N-methyl-DL-aspartate (Asp*) a non-hydrolysable analog of aspartate. Error bars represent ± Standard deviation.

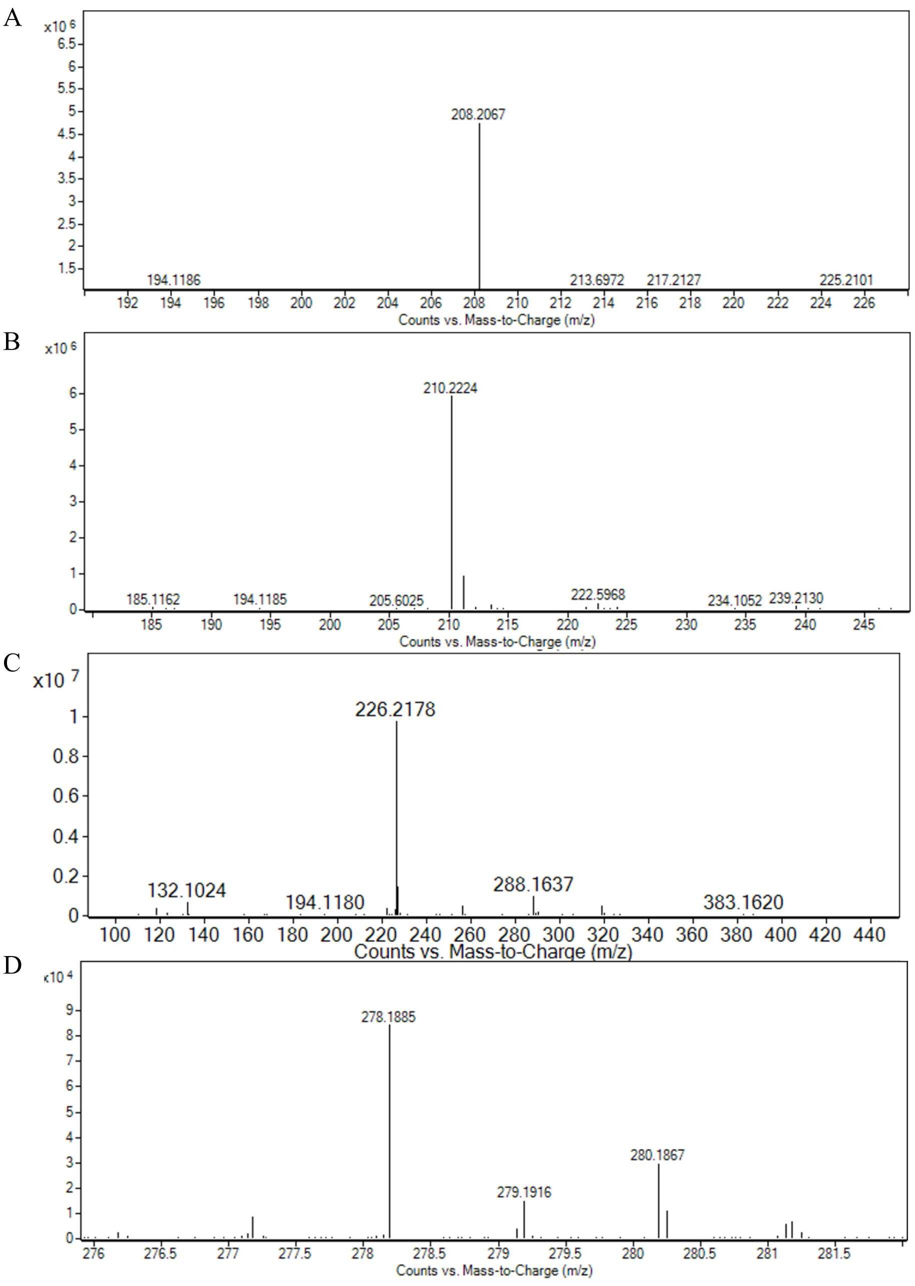

FIG S3. HRESIQTOFMS spectral data of compounds **1-4**

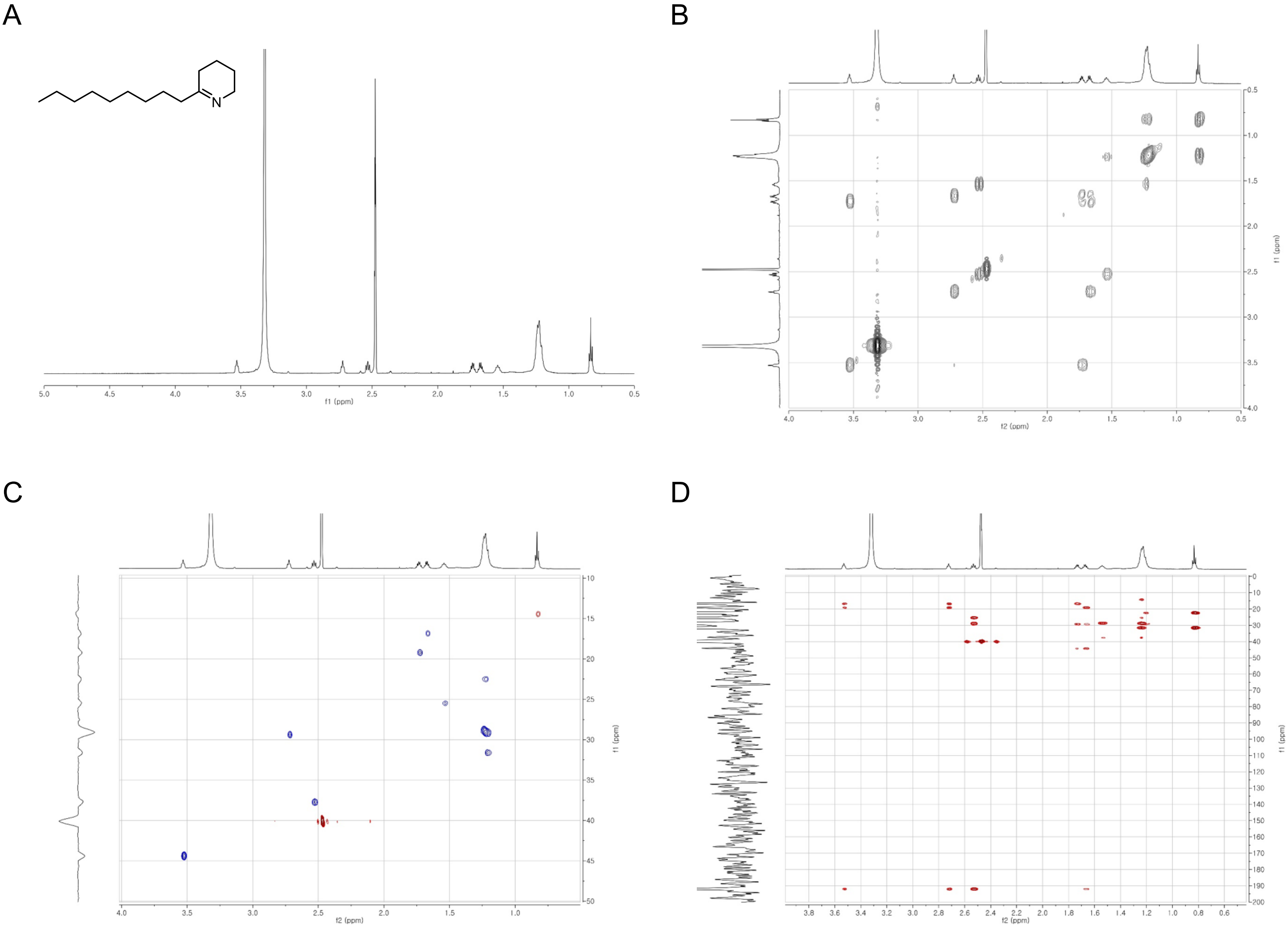

FIG S4. NMR spectra of compound **1**

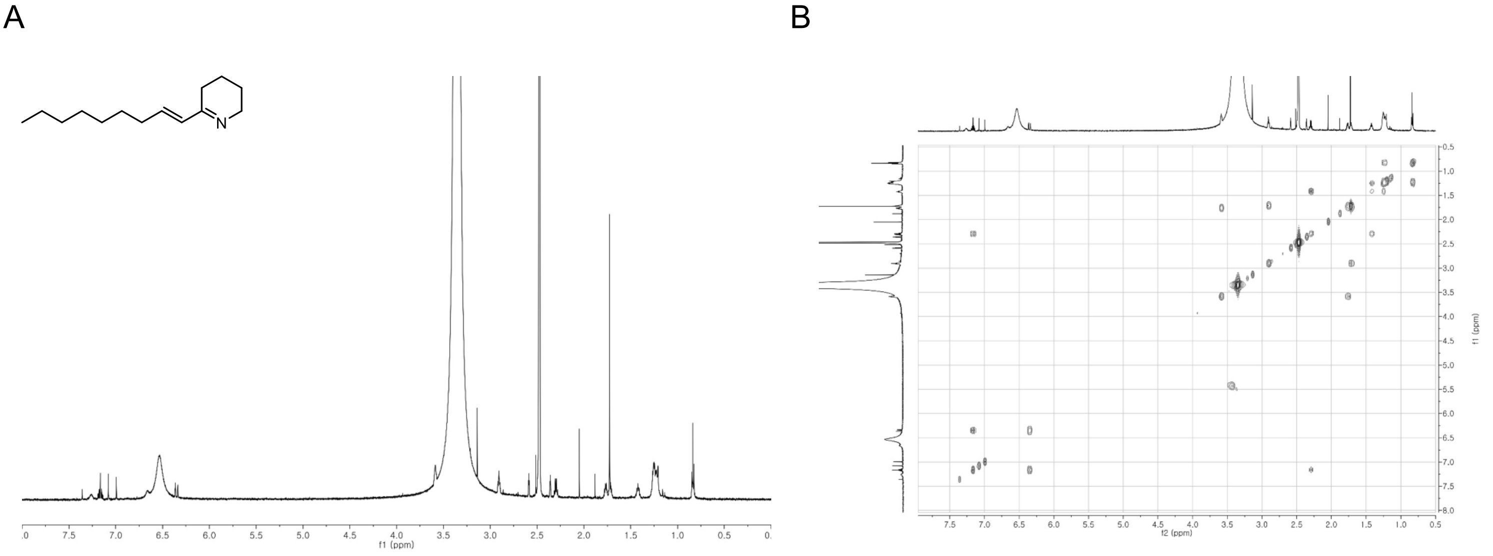

FIG S5. NMR spectra of compound **2**

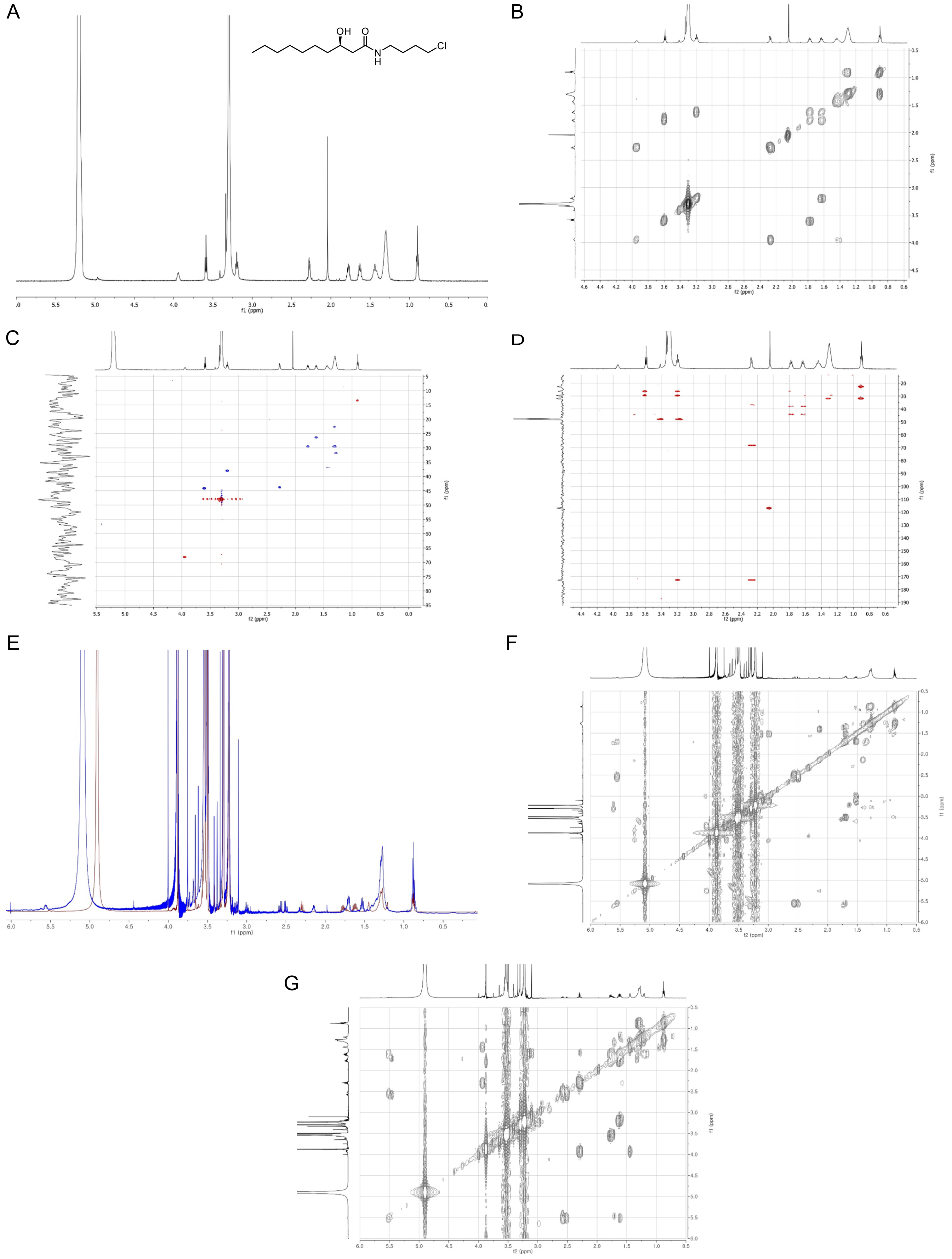

FIG S6. (A-D) NMR spectra of compound **4** (E) 1H NMR spectra comparison of S-MTPA (blue) and R-MTPA (maroon) of compound **4** (F) gCOSY NMR spectrum of S-MTPA of compound **4** (G) gCOSY NMR spectrum of R-MTPA of compound **4**

Compound **1**

Observed MS/MS fragmentation pattern of koreenceine A

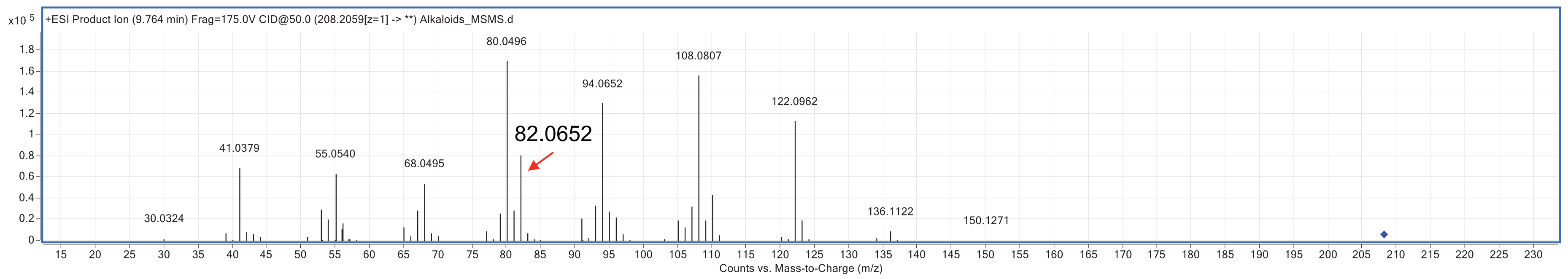

Calculated MS/MS fragmentation pattern of koreenceine A

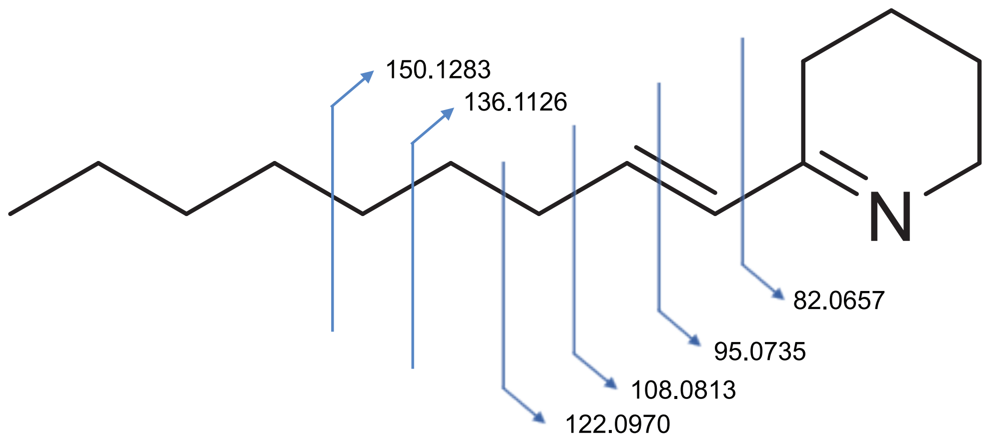

Compound **2**

Observed MS/MS fragmentation pattern of koreenceine B

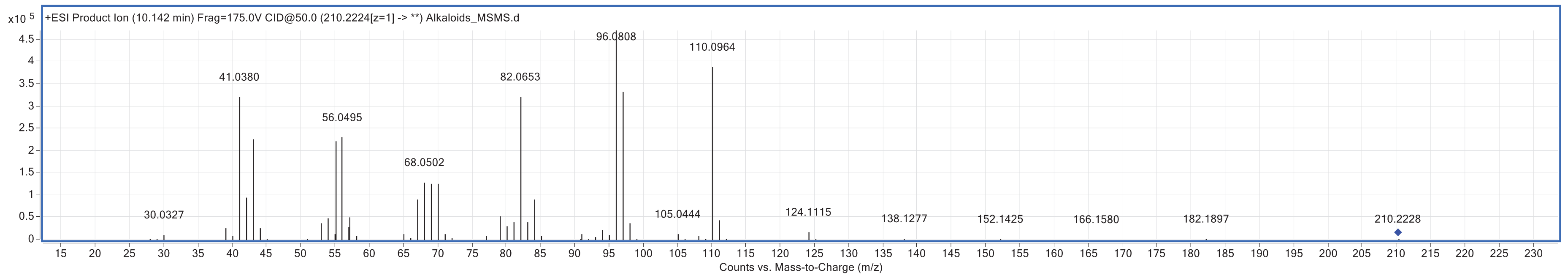

Calculated MS/MS fragmentation pattern of koreenceine B

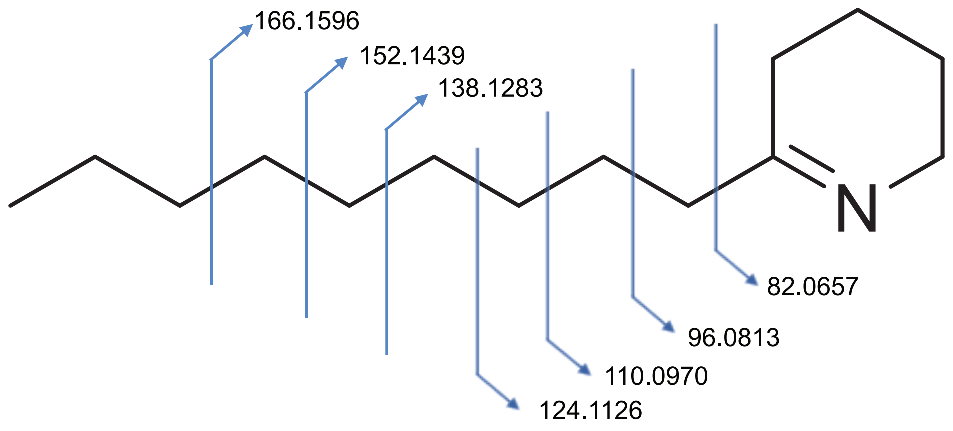

Compound **3**

Observed MS/MS fragmentation pattern of koreenceine C

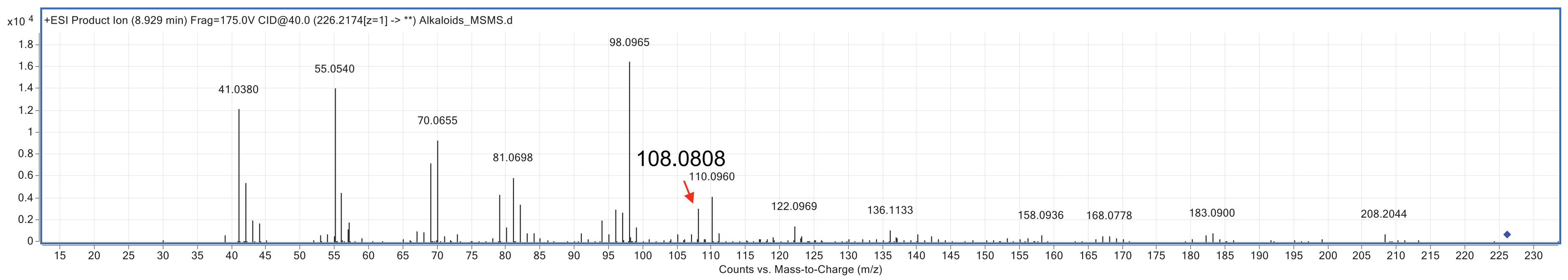

Calculated MS/MS fragmentation pattern of koreenceine C

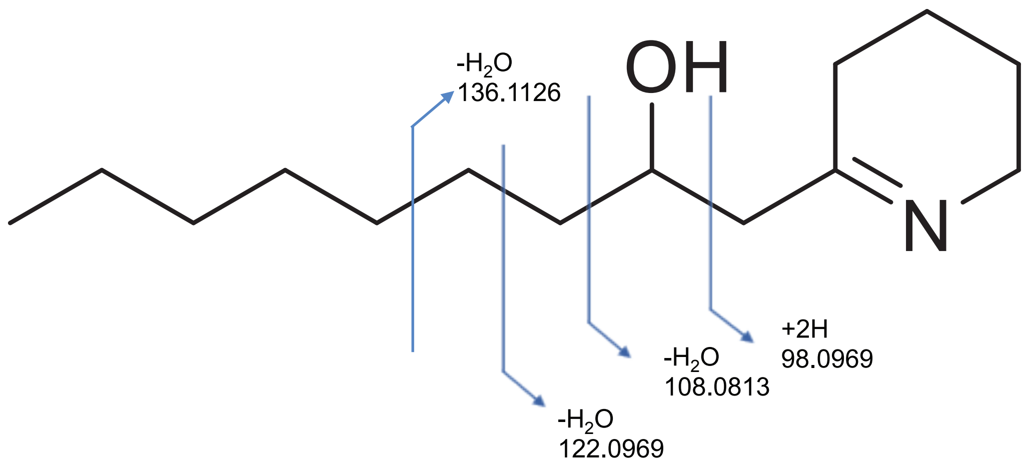

Compound **4**

Observed MS/MS fragmentation pattern of koreenceine D

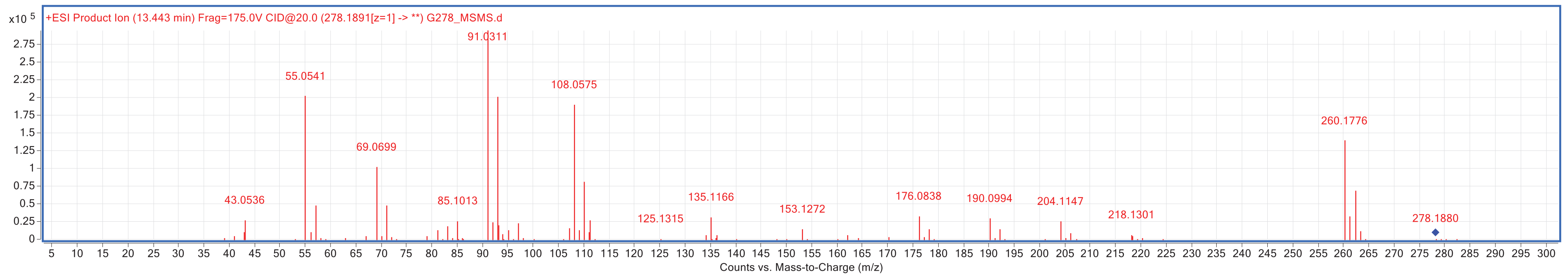

Calculated MS/MS fragmentation pattern of koreenceine D

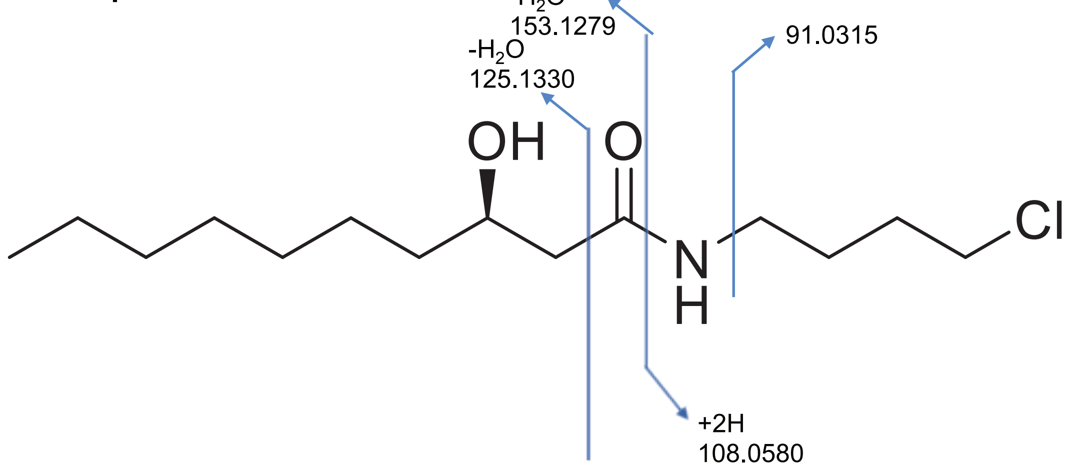

FIG S7. Tandem MS spectra of compounds **1-4**

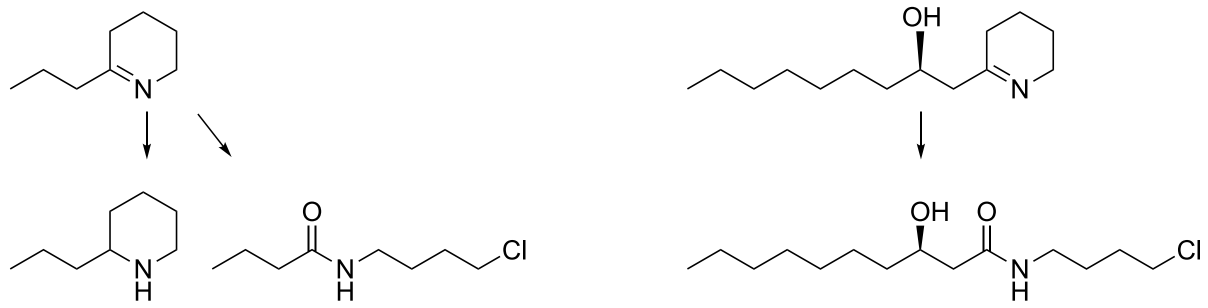

FIG S8. Predicted uncharacterized halogenation reaction and oxidation/reduction of γ-coniceine and koreenceine C.

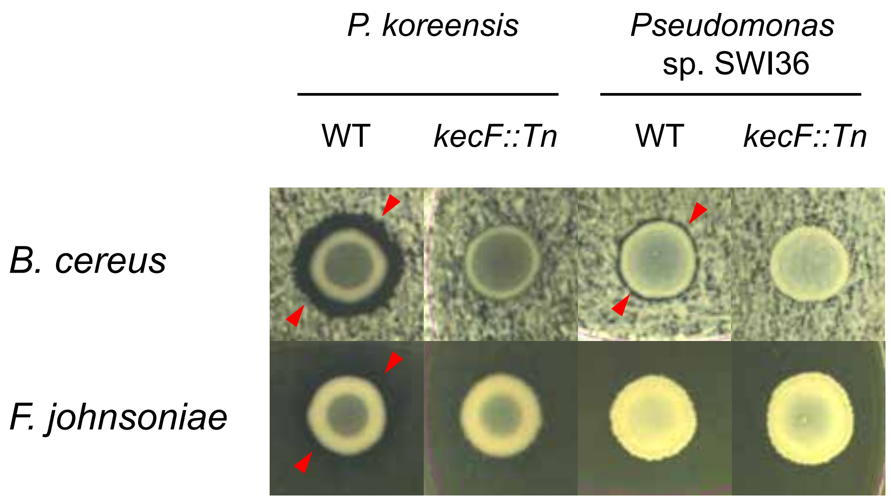

FIG S9. Activity profile of *P. koreensis* and *Pseudomonas* sp. SWI36 against *B. cereus* and *F. johnsoniae*.

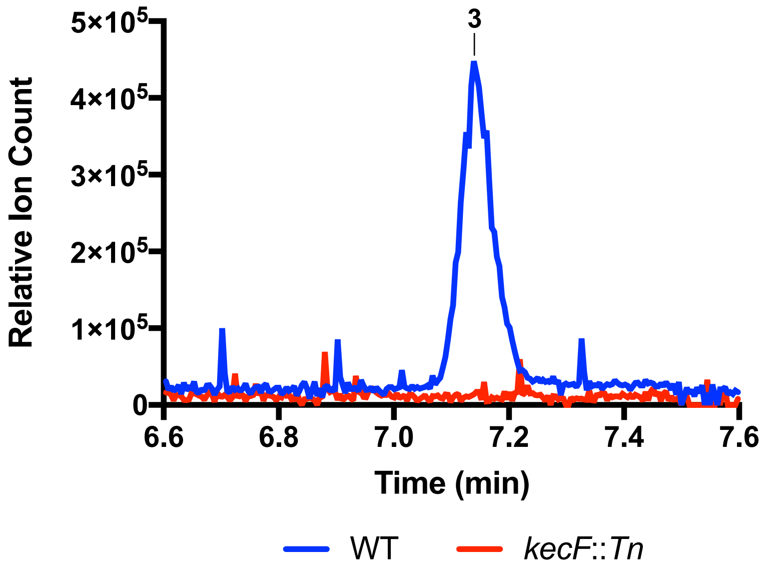

FIG S10. Extracted ion chromatograms of koreenceine C for *Pseudomonas* sp SWI36 wild type and *kecF*::*Tn* mutant.

Table S1. Bacterial genomes containing gene clusters with high similarity to the koreenceine cluster.

Name	Abbreviation	GenBank assembly accession	Koreenceine-like cluster clade
[Flavobacterium] sp. 29	29	GCA_002754355	A
Pseudomonas asplenii 4A7	4A7	GCA_002891515	A
Pseudomonas baetica a390	a390	GCA_003031005	A
Pseudomonas baetica LMG 25716	LMG25716	GCA_002813455	A
Pseudomonas fluorescens NZ011	NZ011	GCA_000276585	A
Pseudomonas fluorescens BW11P2	BW11P2	GCA_001679645	A
Pseudomonas fluorescens C3	C3	GCA_000967955	A
Pseudomonas fluorescens H16	H16	GCA_000802985	A
Pseudomonas fluorescens MS82	MS82	GCA_003055645	A
Pseudomonas jessenii LBp-160603	LBp160603	GCA_003205375	A
Pseudomonas koreensis CI12	CI12	GCA_002003425	A
Pseudomonas mandelii JR-1	JR1	GCA_000257545	A
Pseudomonas mandelii PD30	PD30	GCA_000690555	A
Pseudomonas mandelii LMG 21607	LMG21607	GCA_900106065	A
Pseudomonas prosekii LMG 26867	LMG26867	GCA_900105155	A
Pseudomonas prosekii P2406	P2406	GCA_003122265	A
Pseudomonas prosekii P2673	P2673	GCA_003122305	A
Pseudomonas sp. 31-12	3112	GCA_003151075	A
Pseudomonas sp. 43NM1	43NM1	GCA_002836905	A
Pseudomonas sp. AD21	AD21	GCA_002878485	A
Pseudomonas sp. B15(2017)	B152017	GCA_002113215	A
Pseudomonas sp. B17(2017)	B172017	GCA_002113765	A
Pseudomonas sp. B2(2017)	B22017	GCA_002113685	A
Pseudomonas sp. B20(2017)	B202017	GCA_002113125	A
Pseudomonas sp. B26(2017)	B262017	GCA_002113045	A
Pseudomonas sp. B35(2017)	B352017	GCA_002113645	A
Pseudomonas sp. B7(2017)	B72017	GCA_002112745	A
Pseudomonas sp. FSL W5-0299	FSLW50299	GCA_002005125	A
Pseudomonas sp. FW305-BF8	FW305BF8	GCA_002883215	A
Pseudomonas sp. GM16	GM16	GCA_000282155	A
Pseudomonas sp. GM24	GM24	GCA_000282235	A
Pseudomonas sp. GV091	GV091	GCA_003053805	A
Pseudomonas sp. Irchel s3a12	Irchels3a12	GCA_900187485	A
Pseudomonas sp. Irchel s3f10	Irchels3f10	GCA_900187515	A
Pseudomonas sp. Irchel s3h9	Irchels3h9	GCA_900187475	A

Pseudomonas sp. MPR-E5	MPRE5	GCA_002883015	A
Pseudomonas sp. MS586	MS586	GCA_001594225	A
Pseudomonas sp. Ok266	ok266	GCA_900110195	A
Pseudomonas sp. OV221	OV221	GCA_003391605	A
Pseudomonas sp. OV657	OV657	GCA_003386585	A
Pseudomonas sp. R15(2017)	R152017	GCA_002112685	A
Pseudomonas sp. R23(2017)	R232017	GCA_002112575	A
Pseudomonas sp. R24(2017)	R242017	GCA_002113465	A
Pseudomonas sp. R27(2017)	R272017	GCA_002113445	A
Pseudomonas sp. Root329	Root329	GCA_001424925	A
Pseudomonas fluorescens BS2	BS2	GCA_000308175	B
Pseudomonas fluorescens AHK-1	AHK1	GCA_003363095	B
Pseudomonas fluorescens FW300-N1B4	FW300N1B4	GCA_001625455	B
Pseudomonas hunanensis P11	P11	GCA_002910975	B
Pseudomonas marginalis BS2952	BS2952	GCA_900105325	B
Pseudomonas monteilii SB3078	SB3078	GCA_000510285	B
Pseudomonas monteilii SB3101	SB3101	GCA_000510325	B
Pseudomonas monteilii MO2	MO2	GCA_001571445	B
Pseudomonas plecoglossicida DJ-1	DJ1	GCA_002307455	B
Pseudomonas plecoglossicida MR134	MR134	GCA_002864885	B
Pseudomonas plecoglossicida MR135	MR135	GCA_002864795	B
Pseudomonas plecoglossicida MR170	MR170	GCA_002864845	B
Pseudomonas plecoglossicida MR69	MR69	GCA_002864775	B
Pseudomonas plecoglossicida MR70	MR70	GCA_002864905	B
Pseudomonas plecoglossicida MR83	MR83	GCA_002864865	B
Pseudomonas plecoglossicida NyZ12	NyZ12	GCA_000831585	B
Pseudomonas plecoglossicida TND35	TND35	GCA_000764405	B
Pseudomonas putida B6-2	B62	GCA_000226035	B
Pseudomonas putida BIRD-1	BIRD1	GCA_000183645	B
Pseudomonas putida F1	F1	GCA_000016865	B
Pseudomonas putida HB3267	HB3267	GCA_000325725	B
Pseudomonas putida JB	JB	GCA_001767335	B
Pseudomonas putida KT2440	KT2440	GCA_000007565	B
Pseudomonas putida LS46	LS46	GCA_000294445	B
Pseudomonas putida ND6	ND6	GCA_000264665	B
Pseudomonas putida S12	S12	GCA_000495455	B
Pseudomonas putida SJTE-1	SJTE1	GCA_000271965	B
Pseudomonas putida Idaho	Idaho	GCA_000226475	B
Pseudomonas putida CA-3	CA3	GCA_002810225	B

Pseudomonas putida DPA1	DPA1	GCA_002891885	B
Pseudomonas putida strain DZ-C18	DZC18	GCA_002094795	B
Pseudomonas putida FDAARGOS_409	FDAARGOS409	GCA_002554535	B
Pseudomonas putida H	H	GCA_001077495	B
Pseudomonas putida HB13667	HB13667	GCA_001306495	B
Pseudomonas putida INSali382	INSali382	GCA_001653615	B
Pseudomonas putida JLR11	JLR11	GCA_001183585	B
Pseudomonas putida KCJK7911	KCJK7911	GCA_003053335	B
Pseudomonas putida N1R	N1R	GCA_900156185	B
Pseudomonas putida P1	P1	GCA_001865225	B
Pseudomonas putida PD1	PD1	GCA_000799625	B
Pseudomonas putida SF1	SF1	GCA_001027965	B
Pseudomonas putida UV4	UV4	GCA_002165695	B
Pseudomonas putida UV4/95	UV495	GCA_002165665	B
Pseudomonas putida TRO1	TRO1	GCA_000367825	B
Pseudomonas taiwanensis SJ9	SJ9	GCA_000500605	B
Pseudomonas sp. 22 E 5	22E5	GCA_900004705	B
Pseudomonas sp. 2822-17	282217	GCA_002742485	B
Pseudomonas sp. 2995-1	29951	GCA_002742505	B
Pseudomonas sp. 31 E 5	31E5	GCA_900005815	B
Pseudomonas sp. 31 E 6	31E6	GCA_900005935	B
Pseudomonas sp. B12(2017)	B122017	GCA_002113785	B
Pseudomonas sp. B13(2017)	B132017	GCA_002113245	B
Pseudomonas sp. B14(2017)	B142017	GCA_002113255	B
Pseudomonas sp. B22(2017)	B222017	GCA_002113105	B
Pseudomonas sp. B23(2017)	B232017	GCA_002113725	B
Pseudomonas sp. B24(2017)	B242017	GCA_002113085	B
Pseudomonas sp. B28(2017)	B282017	GCA_002113025	B
Pseudomonas sp. B4(2017)	B42017	GCA_002113565	B
Pseudomonas sp. B8(2017)	B82017	GCA_002113575	B
Pseudomonas sp. C5pp	C5pp	GCA_000814065	B
Pseudomonas sp. CC6-YY-74	CC6YY74	GCA_002025205	B
Pseudomonas sp. FFUP_PS_41	FFUPPS41	GCA_002858645	B
Pseudomonas sp. FW305-E2	FW305E2	GCA_002901725	B
Pseudomonas sp. GTC 16473	GTC16473	GCA_001753855	B
Pseudomonas sp. GTC 16482	GTC16482	GCA_001319995	B
Pseudomonas sp. Irchel 3H9	Irchel3H9	GCA_900187495	B
Pseudomonas sp. JY-Q	JYQ	GCA_001655295	B
Pseudomonas sp. Leaf58	Leaf58	GCA_001422615	B

Pseudomonas sp. LG1E9	LG1E9	GCA_003290225	B
Pseudomonas sp. MIACH	MIACH	GCA_001269925	B
Pseudomonas sp. MR 02	MR02	GCA_002797475	B
Pseudomonas sp. NBRC 111118	NBRC111118	GCA_001320085	B
Pseudomonas sp. NBRC 111121	NBRC111121	GCA_001320165	B
Pseudomonas sp. NBRC 111125	NBRC111125	GCA_001320295	B
Pseudomonas sp. NBRC 111136	NBRC111136	GCA_001320745	B
Pseudomonas sp. NBRC 111139	NBRC111139	GCA_001753955	B
Pseudomonas sp. OV577	OV577	GCA_003386595	B
Pseudomonas sp. P21	p21	GCA_001642705	B
Pseudomonas sp. PGPPP2	PGPPP2	GCA_002255825	B
Pseudomonas sp. RIT357	RIT357	GCA_000632245	B
Pseudomonas sp. RW405	RW405	GCA_003184135	B
Pseudomonas sp. SID14000	SID14000	GCA_002165135	B
Pseudomonas sp. SMT-1	SMT1	GCA_003204195	B
Pseudomonas sp. SWI36	SWI36	GCA_002948105	B
Pseudomonas sp. XWY-1	XWY1	GCA_002953115	B
Streptomyces albulus CCRC 11814	CCRC11814	GCA_000403765	C
Streptomyces albulus NK660	NK660	GCA_000695235	C
Streptomyces albulus PD-1	SaPD1	GCA_000504065	C
Streptomyces albus ZpM	ZpM	GCA_000963515	C
Streptomyces diastatochromogenes NRRL B-1698	NRRLB1698	GCA_001418405	C
Streptomyces noursei ATCC 11455	ATCC11455	GCA_001704275	C
Streptomyces yunnanensis CGMCC 4.3555	CGMCC43555	GCA_900142595	C
Streptomyces sp. MspMP-M5	MspMPM5	GCA_000373585	C
Streptomyces sp. NRRL F-4489	NRRLF4489	GCA_001509485	C
Xenorhabdus bovienii CS03	CS031	GCA_000973125	D
Xenorhabdus bovienii CS03	CS032	GCA_000973125	D
Xenorhabdus bovienii feltiae Florida	Florida	GCA_000736675	D
Xenorhabdus bovienii feltiae France	France	GCA_000736655	D
Xenorhabdus bovienii feltiae Moldova	Moldova	GCA_000736595	D
Xenorhabdus bovienii intermedium	intermedium	GCA_000736615	D
Xenorhabdus bovienii kraussei	kraussei	GCA_00073669	D
Xenorhabdus bovienii kraussei Quebec	Quebec	GCA_000736555	D
Xenorhabdus bovienii oregonense	oregonense	GCA_000736535	D
Xenorhabdus bovienii puntauvense	puntauvense	GCA_000736635	D
Xenorhabdus khoisanae MCB sp. ICMP 17674	ICMP17674	GCA_001037465	D
Xenorhabdus poinarii G6	G6	GCA_000968175	D

Xenorhabdus sp. NBAII XenSa04	XenSa04	GCA_000798625	D
Uncultured bacterium	pEAF66	EU099626	
